# Multi-photon attenuation-compensated light-sheet fluorescence microscopy

**DOI:** 10.1038/s41598-020-64891-8

**Published:** 2020-05-15

**Authors:** Madhu Veettikazhy, Jonathan Nylk, Federico Gasparoli, Adrià Escobet-Montalbán, Anders Kragh Hansen, Dominik Marti, Peter Eskil Andersen, Kishan Dholakia

**Affiliations:** 1 DTU Health Tech, Technical University of Denmark, Frederiksborgvej 399, 4000 Roskilde, Denmark; 2 SUPA, School of Physics and Astronomy, University of St Andrews, North Haugh, St Andrews, KY16 9SS UK; 3 DTU Fotonik, Technical University of Denmark, Frederiksborgvej 399, 4000 Roskilde, Denmark; 4 Department of Physics, College of Science, Yonsei University, Seoul, 03722 South Korea; 5 Present Address: School of Science and Engineering, University of Dundee, Dundee, DD1 4HN UK

**Keywords:** Optics and photonics, Microscopy, Light-sheet microscopy, Multiphoton microscopy, Cellular imaging

## Abstract

Attenuation of optical fields owing to scattering and absorption limits the penetration depth for imaging. Whilst aberration correction may be used, this is difficult to implement over a large field-of-view in heterogeneous tissue. Attenuation-compensation allows tailoring of the maximum lobe of a propagation-invariant light field and promises an increase in depth penetration for imaging. Here we show this promising approach may be implemented in multi-photon (two-photon) light-sheet fluorescence microscopy and, furthermore, can be achieved in a facile manner utilizing a graded neutral density filter, circumventing the need for complex beam shaping apparatus. A “gold standard” system utilizing a spatial light modulator for beam shaping is used to benchmark our implementation. The approach will open up enhanced depth penetration in light-sheet imaging to a wide range of end users.

## Introduction

Light-sheet fluorescence microscopy (LSFM) has transformed the field of imaging in recent years, owing to its optical sectioning capabilities, resulting in fast, highly resolved images with significantly reduced photo-bleaching and photo-toxicity^1,2^. Propagation-invariant light fields, such as Airy and Bessel beams, have been employed in LSFM not only because of their pseudo-nondiffracting properties which enables them to retain their transverse profile on propagation, but also due to their self-healing capabilities on interaction with obstacles during propagation^3–7^. However, attenuation due to scattering and absorption results in an exponential decay of intensity of any given optical field as it penetrates deep into tissue and limits the penetration depth achievable for deep tissue imaging. Recently, the capability to shape the envelope profile of a light field arbitrarily^8–11^ has been demonstrated to counteract the attenuation-induced exponential decrease in intensity, by tailoring an exponential rise in intensity along the direction of propagation^10^. Building upon this, LSFM exploiting attenuation-compensated Airy beams in single-photon imaging has demonstrated improved image quality at depth in attenuating biological specimens without any increase in the peak intensity of the illuminating light-sheet^12^. This is achieved by the selective delivery of additional intensity to greater depths within the attenuating medium, potentially minimizing photo-damage across the specimen. Our previous work utilized a spatial light modulator (SLM) for generation of attenuation-compensated Airy beams solely for single-photon imaging. While this approach offers excellent beam quality and has the flexibility to dynamically adjust the beam shape to optimally counteract the specimen attenuation, it adds cost and complexity to such a system, limiting the potential uptake of the method.

In this work, we show that attenuation-compensation of propagation invariant Airy fields can be achieved for multi-photon (two-photon) LSFM, and can be implemented in an inexpensive and facile manner. This is achieved by exploiting readily-available graded neutral density filters (NDF), effectively eliminating the need for an SLM. Increases in signal-to-noise ratio (SNR) of up to 45% with NDF-based two-photon attenuation-compensated Airy LSFM and 65% with NDF-based single-photon attenuation-compensated Airy LSFM are observed in biological specimens.

## Results

**Figure 1:**
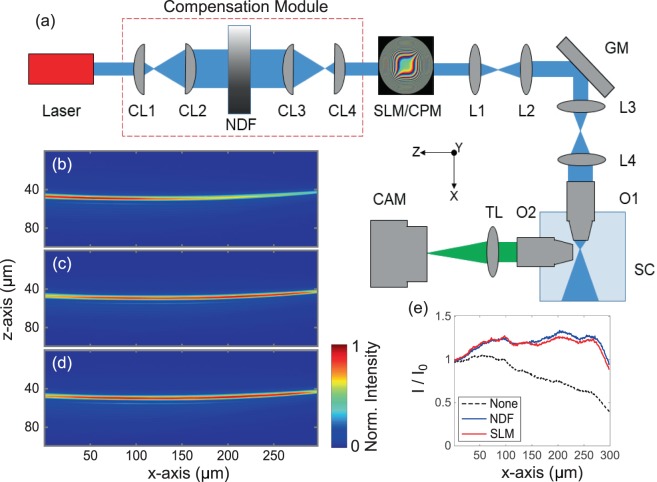
Experimental design of the attenuation-compensated Airy light sheet microscope, and Airy beam profiles. (**a**) Experiment setup with attenuation-compensation provided by the NDF. (**b**–**d**) show the 2 + 1D Airy light-sheet profiles attenuated by an absorbing NIR dye (2 mM) and visualized in fluorescein to show two-photon fluorescence with (**b**) no attenuation-compensation, (**c**) attenuation-compensated by the NDF, and (**d**) attenuation-compensated by the SLM ($\sigma =0.5$). (**e**) Normalized peak transverse intensity versus propagation distance corresponding to beam profiles in (b–d). CAM: Camera, CL: Cylindrical Lens, CPM: Cubic Phase Mask, GM: Galvo Mirror, L: Lens, NDF: Neutral Density Filter, O: Objective, SC: Sample Chamber, SLM: Spatial Light Modulator, TL: Tube Lens. The $x,y,z$ coordinate system shown in (a) applies within the sample volume only.

The cylindrical pupil function of an Airy light-sheet, compensated to overcome exponential intensity decay is represented as^12^

1 $$P(u)={A}_{\sigma }\,\exp \mathrm{(2}\pi i\alpha {u}^{3})\,\exp (\sigma [u-1])\,\gamma (u)$$

where $u$ is the normalized pupil coordinate corresponding to the $z-$axis of the microscope (see Fig. 1(a)), ${A}_{\sigma }$ is a real scaling factor, $\alpha$ controls the propagation-invariance of the Airy light-sheet^5^, $\sigma$ dictates the degree of linear attenuation-compensation and the light-sheet propagates in the positive $x$ direction. The first exponential term in Eq. (1) describes the phase modulation required to generate an Airy beam and the second exponent is the amplitude modulation required to combat attenuation. The NDF used in this study (Thorlabs, NDL-25C-4, Optical density: 0.04–4.0) has a neutral density which varies linearly along its length, therefore the transmission through the NDF varies exponentially along its length. $\gamma (u)$ is a smoothly varying apodization function, the exact form of which is not critical. In ref. ^12^, an SLM was used to set $\gamma (u)=\exp (-{u}^{8})\,H(\sqrt{2}-|u|)$, where $H\mathrm{(.)}$ is the Heaviside step function, with an expanded beam. Here, to conserve laser power, we allow the natural Gaussian envelope of the laser to act as the apodization function.

We first tested the fidelity of attenuation-compensated Airy beams generated by the NDF-based approach. Airy light-sheet profiles in the presence of attenuation provided by an absorbing NIR dye (American Dye Source, Inc., ADS795WS, absorption coefficient: $1.6\times {10}^{5}\,$L mol^−1^ cm^−1^, 2 mM) were visualized in fluorescein. Figure 1(b) shows the Airy light-sheet profile with no attenuation-compensation. Figure 1(c,d) show the Airy light-sheet with NDF-based and SLM-based ($\sigma =0.5$) attenuation-compensation respectively. The peak intensity in the planes transverse to beam propagation ($y-z$ plane), normalized to the value at $x=0$, as a function of longitudinal coordinate ($x-$axis) (Fig. 1(e)) clearly shows the intensity decay in the main lobe of the Airy beam without attenuation-compensation. We recover a nearly uniform intensity along the full extent of the light-sheet using either the NDF- or SLM-based compensation techniques.

Absorption and scattering are two key phenomena that impede deeper penetration of incident optical fields into tissue. While absorption and single-scattering yield exponential decay of the incident light intensity described by the Beer-Lambert law, the increasing contribution of multiple scattering with deeper penetration into the sample may yield strong deviations from the expected exponential decay. Prior studies on attenuation-compensation have only considered compensation of an exponential decay. However, it is possible to control the intensity evolution of the beam arbitrarily, and the decay profile may be compensated for with sufficient characterization of the specimen. Using MCmatlab^13^, an open-source Monte Carlo radiative transport program, we found that the intensity decay of the incident light field followed an exponential profile even in the presence of high scattering anisotropy (see Supplementary Note 1). This result means that compensation of the exponential intensity decay of light is sufficient, and arbitrary control of the intensity evolution of the beam is not required, for a wide range of specimens. Therefore, we are able to use a standard optical element, the NDF, for providing attenuation-compensation of the field.

### Two-Photon Airy LSFM Imaging Results

**Figure 2:**
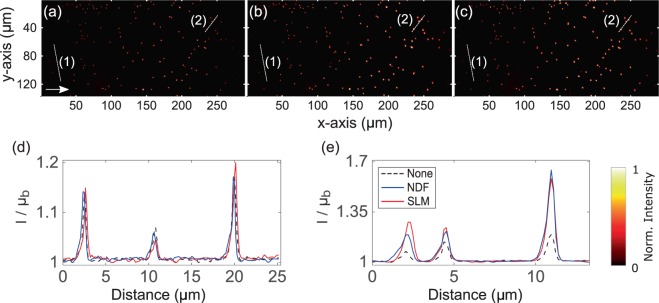
Maximum intensity projections of the recorded two-photon Airy LSFM images (**a**–**c**) of 400 nm diameter fluorescent microspheres in attenuating 2 mM concentrated NIR dye solution. (**a**) No attenuation-compensation, (**b**) attenuation-compensation using NDF, and (**c**) attenuation-compensation using SLM ($\sigma =0.5$). (**d**) Intensity profiles along the dashed line (1) shown in (**a**–**c**). (**e**) Intensity profiles along the dashed line (2) shown in (**a**–**c**) at a depth of ~$230\,\mu m$ into the sample. The arrow in (**a**) denotes the direction of propagation of the Airy light-sheet. Look-up tables of the images shown in (**a**–**c**) are independently scaled to the data shown. Line intensity profiles in (**d**,**e**) are normalized with respect to the local mean background ${{\rm{\mu }}}_{{\rm{b}}}$.

The two-photon Airy LSFM was set up to directly compare the image quality achieved between NDF- and SLM-based attenuation-compensation methods. A 3D suspension of 400 nm diameter green fluorescent beads was made in a $\mathrm{1 \% }$ agarose gel, containing $2$ mM NIR dye, to yield attenuation by absorption. Figure 2 shows maximum intensity projections of the recorded images of these samples and the normalized line intensity profiles taken through them, showing an enhancement in signal-to-background ratio (SBR) at depth when attenuation-compensation is used. Both NDF- and SLM-based methods achieved similar enhancements.

**Figure 3:**
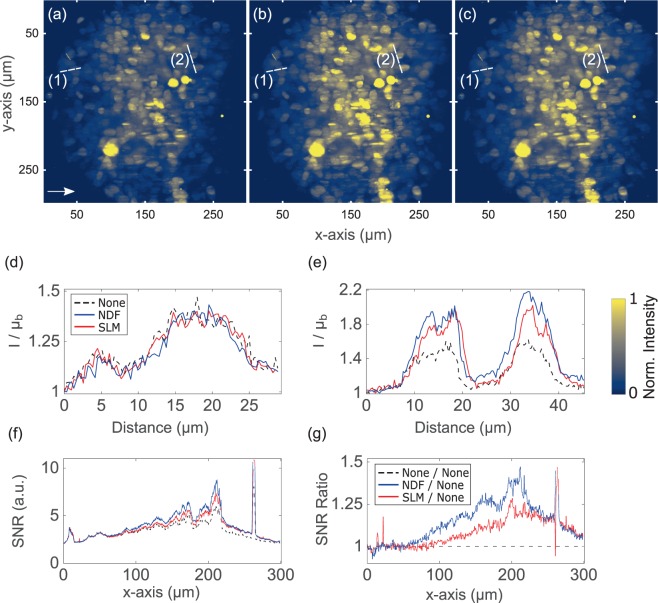
Maximum intensity projections of recorded two-photon Airy LSFM images (**a**–**c**) of $300$ μm diameter HEK-293 spheroid stably expressing GFP. (**a**) No attenuation-compensation, (**b**) attenuation-compensation using NDF, and (**c**) attenuation-compensation using SLM ($\sigma =0.5$). (**d,e**) Intensity profiles along dashed lines (1) and (2) respectively, shown in (**a**–**c**). (**f**) SNR plotted as a function of propagation coordinate, $x$, for no-attenuation compensation, attenuation-compensation using NDF, and attenuation-compensation using SLM ($\sigma =0.5$). (**g**) Ratios of SNR with NDF-based compensation (blue) and SLM-based compensation (red) to no compensation. The arrow in (**a**) denotes the direction of propagation of the Airy light-sheet. Look-up tables of the images shown in (**a**–**c**) are independently scaled to the data shown. Line intensity profiles in (**d**–**e**) are normalized with respect to the local mean background ${{\rm{\mu }}}_{{\rm{b}}}$. This dataset can be accessed at this website^21^.

We further performed a comparison between attenuation-compensation methods in thick biological specimens of diameter between 300 μm–450 μm exhibiting attenuation from both absorption and scattering. Human embryonic kidney 293 (HEK-293) spheroids stably expressing Green Fluorescent Protein (GFP) were imaged. These samples were then fixed in $\mathrm{4 \% }$ paraformaldehyde and embedded in $\mathrm{1 \% }$ agarose gel for imaging. Figure 3(a–c) show images acquired with each method and intensity profiles through lines (1) and (2) are shown in Fig. 3(d,e). We performed a SNR analysis of these 3D image stacks in the spatial frequency domain, taking the Fourier transform of each $y-z$ plane as a function of depth ($x-$axis) into the specimen^6^. We identified spectral bands between ${f}_{r}\mathrm{=10}-\mathrm{50 \% (2}$NA$/\lambda )$ and ${f}_{r}\mathrm{=80}-\mathrm{100 \% (2}$NA$/\lambda )$ corresponding to “signal” and “noise” respectively, and summed the spectral magnitudes within these bands (see Supplementary Note 2 for more details). The trend in SNR across the FOV is shown for each method in Fig. 3(f), and the ratio of SNR in NDF- and SLM-based compensation relative to the case of no compensation is shown in Fig. 3(g). At a depth of $200$ μm, these plots consistently show increases in SNR of ~39% with NDF- and ~27% with SLM-based attenuation-compensation in biological specimen, yielding similar image quality. Besides, a total of 8 HEK-293 spheroid image stacks were acquired, and increases in SNR between $15-\,\mathrm{45 \% }$ and $5-\,\mathrm{25 \% }$ in the NDF- and SLM-based attenuation-compensation cases were observed at a depth of $200$ μm. The single bright spot observed at ~$263$ μm in Fig. 3(a–c) was due to an exceptional expression of GFP of a cell in the spheroid which manifests as a large spike in the SNR ratios as seen in Fig. 3(g). This spot was discarded while estimating the range of enhancement in SNR for the compensation schemes.

### Single-Photon Airy LSFM Imaging Results

**Figure 4:**
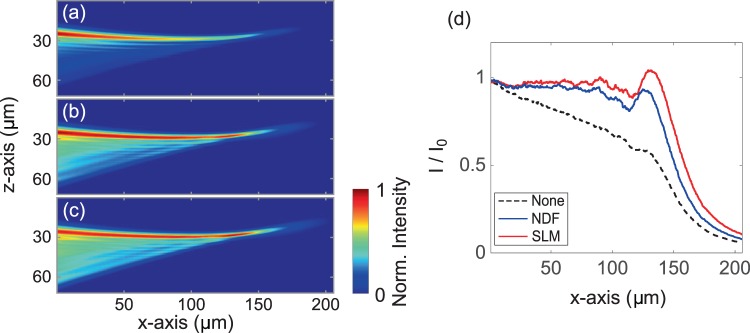
(**a**–**c**) show the 1+1D Airy light-sheet profiles visualized in fluorescein (0.88 mM) to show fluorescence and provide attenuation. (**a**) No attenuation-compensation, (**b**) attenuation-compensated by NDF, and (**c**) attenuation-compensated by SLM ($\sigma =0.8$). (**d**) Normalized peak transverse intensity versus propagation distance corresponding to beam profiles in (**a**–**c**).

In addition, we also investigated the performance of attenuation-compensation in the single-photon excitation regime. The 1+1D Airy light-sheet profiles in the presence of attenuation were visualized in high concentration fluorescein dye (0.88 mM). Figure 4(a–c) show the corresponding Airy profiles with no attenuation-compensation, NDF-based, and SLM-based ($\sigma =0.8$) attenuation-compensation respectively. Uniform intensity in the Airy main caustic after attenuation-compensation can clearly be seen from Fig. 4(b,c) in contrast to the non-compensated case in Fig. 4(a). The analogous longitudinal intensity envelopes measured in the above three cases up to an imaging depth of $200$ μm are shown in Fig. 4(d). The difference in area under the curves in this figure qualitatively represents the level of attenuation-compensation provided by both NDF and SLM over their non-compensated counterpart.

**Figure 5:**
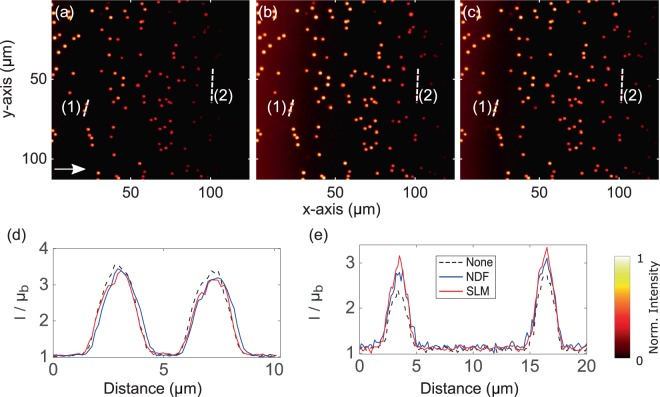
Maximum intensity projections of the deconvolved single-photon Airy LSFM images (**a**–**c**) of $2$ μm diameter fluorescent microspheres in attenuating 0.88 mM concentrated fluorescein solution. (**a**) No attenuation-compensation, (**b**) attenuation-compensation using the NDF, and (**c**) attenuation-compensation using the SLM ($\sigma =0.8$). (**d**) Intensity profiles along the dashed line (1) near the start of the FOV where intensities of all three cases match shown in (**a**–**c**). (**e**) Intensity profiles along the dashed line (2), ~$80$ μm deeper into the sample, shown in (**a**–**c**). The arrow in (**a**) denotes the direction of propagation of the Airy light-sheet. Look-up tables of the images shown in (**a**–**c)** are independently scaled to the data shown. Line intensity profiles in **(d**,**e**) are normalized with respect to the local mean background ${{\rm{\mu }}}_{{\rm{b}}}$.

We made a 3D suspension of 2 μm diameter red fluorescent beads in 1% agarose gel containing 0.88 mM fluorescein, as a phantom acting as an attenuating medium. The attenuation coefficient was determined to be ${C}_{attn}=85.9$ cm^−1^. Figure 5 shows maximum intensity projections of the deconvolved images^5,6^ of these samples and the line profiles taken through them, showing an enhancement in SBR at depth when attenuation-compensation is used. Both NDF- and SLM-based methods achieved similar enhancements.

**Figure 6:**
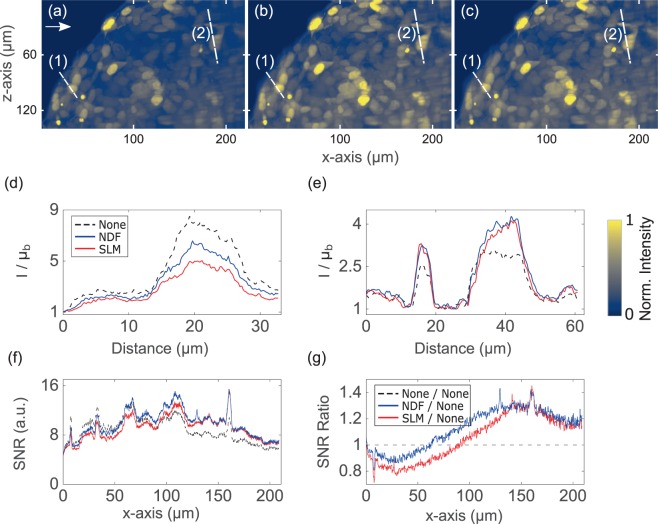
Maximum intensity projections of deconvolved single-photon Airy LSFM images (**a**–**c**) of ~$450$ μm diameter SH-SY5Y spheroid stably expressing GFP. (**a**) No attenuation-compensation, (**b**) attenuation-compensation using NDF, and (**c**) attenuation-compensation using SLM ($\sigma =0.8$). (**d**) Intensity profiles along line (1) near the start of the FOV where intensities of all three cases match and (**e**) intensity profiles along line (2), ~$150\,\mu m$ deeper into the sample. (**f**) SNR plotted as a function of propagation coordinate, $x$, for no-attenuation compensation, attenuation-compensation using NDF, and attenuation-compensation using SLM ($\sigma =0.8$). (**g**) Ratios of SNR with NDF-based and SLM-based compensations compared to the case of no compensation. The arrow in (a) denotes the direction of propagation of the Airy light-sheet. Look-up tables of the images shown in (**a**–**c**) are independently scaled to the data shown. Line intensity profiles in (**d,e**) are normalized with respect to the local mean background ${{\rm{\mu }}}_{{\rm{b}}}$. This dataset can be accessed at^21^.

Finally, we used a biological specimen in the single-photon attenuation-compensated Airy LSFM. Spheroids, comprising of human neuroblastoma (SH-SY5Y) cells stably expressing GFP, were studied. Figure 6 shows the $x-z$ maximum intensity projections of the deconvolved images. Similar to the two-photon case, the spheroid was also imaged under three different conditions: no compensation, NDF-based attenuation-compensation, and SLM-based attenuation-compensation($\sigma =0.8$). Similar to the two-photon scenario, the SNRs for the different compensation schemes were calculated after analysing the Fourier content of the deconvolved image stacks. Figure 6(f) plots the SNR against the propagation coordinate at the areas specified in Fig. 6(a–c,g) plots ratios of the data relative to the non-compensated case. These plots show increases in SNR of ~$\mathrm{30 \% }$ with NDF- and ~$\mathrm{29 \% }$ with SLM-based attenuation-compensation, in biological specimen, at a depth of $140$ μm. Additionally, increases in SNR between 25–50% and 20–40% in the NDF- and SLM-based attenuation-compensation cases were observed at a depth of $140$ μm, from a total of 4 SH-SY5Y spheroid image stacks acquired.

## Discussion

We have utilized attenuation-compensation for Airy beam based LSFM to selectively deliver more light deeper into a specimen without increasing the peak illumination intensity, therefore minimizing photo-damage. We have demonstrated the utility of attenuation-compensation provided by a NDF in LSFM and, for the first time, demonstrated an attenuation-compensated two-photon Airy LSFM system. Attenuation-compensation was applied to a light-sheet using an SLM or an NDF, and the performance of the two methods was compared.

**Figure 7:**
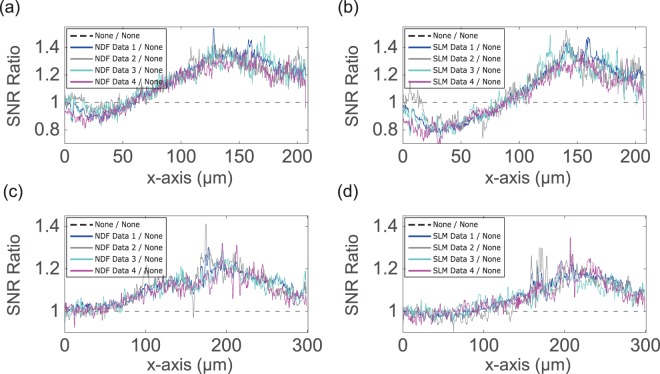
Ratios of SNR with NDF-based compensation and SLM-based compensation to no compensation of **(a,b)** deconvolved single-photon Airy LSFM image stacks of ~450 μm diameter SH-SY5Y spheroid and **(c,d)** recorded two-photon Airy LSFM image stacks of ~450 m diameter HEK-293 spheroid. (**a**,**b**) Data 1 - complete stack, Data 2–15 μm subset of complete stack at $z=75\,\mu m$, Data 3–15 μm subset of complete stack at $z=90\,\mu m$, and Data 4–20 μm subset of complete stack at $z=105\,\mu m$ from the start of the stack. (**c,d**) Data 1 - complete stack, Data 2–15 μm subset of complete stack at $z=25\,\mu m$, Data 3–15 μm subset of complete stack at $z=40\,\mu m$, and Data 4–55 μm subset of complete stack at $z=105\,\mu m$ from the start of the stack. This dataset can be accessed at this website^21^.

Through SNR measurements, we showed an enhanced feature contrast at depth for single- and two-photon Airy LSFM with attenuation-compensation employed using either SLM or NDF. We imaged multiple spheroids of SH-SY5Y and HEK-293 cells with diameter varying between $300\,\mu m-450\,\mu m$. For brevity, only two sets of data, out of these, are presented here (Figs. 3 and 6). In two-photon Airy LSFM, increases in SNR at $200\,\mu m$ deep in the HEK-293 spheroid were observed between $5-\mathrm{150 \% }$ and $5-\mathrm{50 \% }$ in the NDF- and SLM-based attenuation-compensation cases respectively. Similar SNR increases of $15-\mathrm{65 \% }$ and $10-\mathrm{65 \% }$ in the NDF- and SLM-based attenuation-compensation cases respectively at $140\,\mu m$ depth in SH-SY5Y spheroid were observed in single-photon Airy LSFM. The spheroid in Fig. 3 is placed centrally with respect to the FOV, and the constituent cells are evenly distributed throughout the volume. The general increase in brightness near the centre of FOV could be attributed to the fact that there is a higher probability of finding a bright cell near the center since there are more cells along the projection in the middle. Figure 7 shows the sample-dependent improvement in SNR for NDF- and SLM-based attenuation-compensated single- and two-photon Airy LSFM, where the data correspond to eight multiple regions in different spheroid specimens. On average, the SNR corresponding to NDF-based two-photon Airy LSFM images of $450\,\mu m$ diameter HEK-293 spheroid exceeds the non-compensated value by ~$\mathrm{4 \% }$ at a depth of $50\,\mu m$ and then rises approximately linearly to a maximum improvement of ~$\mathrm{22 \% }$ at a depth of 200 m. Similarly, the SNR for SLM-based method mimics the non-compensated value at a depth of 50 m and then rises approximately linearly to a maximum improvement of ~$\mathrm{20 \% }$ at a depth of 200 μm.

We note that, in the single-photon image data (Fig. 6), attenuation-compensation results in a small reduction in SNR near the start of the FOV which was not observed in the two-photon experiments (Fig. 3). When considering the one- and two-photon light-sheet excitation profiles (Figs. 1 and 4), this can be understood by the considerable background present in the sidelobe region of the beam at the start of the FOV when attenuation-compensation is used. This large background component will reduce the contrast of the sidelobes and have an adverse effect on the deconvolution of the single-photon images. However, in two-photon excitation, the non-linear relationship between illumination intensity and fluorescence excitation will suppress this feature. This interesting effect, coupled with the fact that scattering generally reduces with increasing wavelength and absorption becomes the more dominant loss mechanism at the longer wavelengths used for multi-photon microscopy, suggest that attenuation-compensation may be most useful in the multi-photon regime. We have not discussed the detection direction of the fluorescence signal in this work, which also deserves attention as it may become a limitation for imaging at depth, particularly in single-photon LSFM.

It is interesting to consider applying attenuation-compensation to Bessel LSFM in a facile manner. Using a linear NDF would not be ideal in such a system since the required pupil function consists of concentric rings with varying amplitude and phase. This results in a smooth exponentially growing profile as we give a full wave-optics treatment of the problem. An exicon^14,15^ would be the closest solution, but is not an off-the-shelf component. Although one could state that a cubic phase mask is also not an off-the-shelf element, it has an inherent simplicity compared to exicon or a quadratic phase element and thus is more amenable to an NDF.

Attenuation-compensation for an Airy beam relies on the application of both phase and amplitude modulation. By use of an NDF, a well defined amplitude modulation may be applied with the SLM imposing the required cubic phase for the resultant Airy profile. In the case of sole use of a single nematic SLM for both amplitude and phase modulation, we have applied the phase for the Airy profile and assumed a linear amplitude response for the device. However, in most cases this is not the case and a modest nonlinear modulation component is present which ideally would be accounted for by calibration. The absence of this amplitude calibration in our case leads to a small mismatch between the NDF + SLM and SLM-only cases for attenuation-compensation. The difference in SNR at depth in the sample as is seen in the data is attributed to this reason. It also highlights the fact that the NDF system avoids the requirement for amplitude modulation of the SLM adding an advantage to its use for attenuation-compensation.

**Figure 8:**
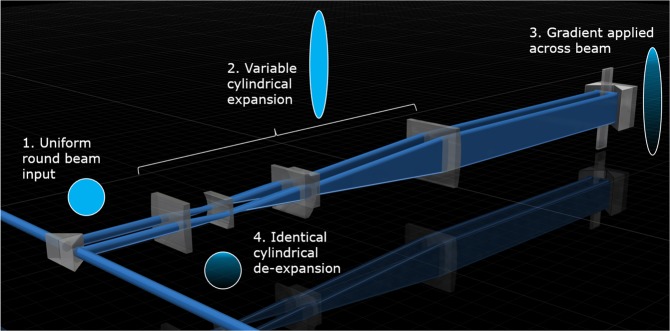
Schematic of the variable attenuation-compensation module based on an NDF and cylindrical zoom lens unit.

The gold-standard approach using an SLM offers dynamic control over the degree of attenuation-compensation. However, in the system we demonstrated, the compensation module in Fig. 1 consisting of an NDF exhibits virtually identical performance with that of the SLM for a fixed set of parameters. Flexibility to change the strength of compensation may be achieved by combining the NDF with cylindrical zoom lenses^16^ for variable (de-)expansion. Figure 8 illustrates this concept, using a double-pass through the cylindrical zoom unit to ensure identical (de-)expansion. Such a system would enable dynamically reconfigurable attenuation-compensation without the need for complex diffractive optics. We expect that our approach will open up enhanced depth penetration using propagation-invariant beams in optical manipulation, optical coherence tomography, extended-depth multi-photon microscopy, and other imaging modalities.

## Methods

### Attenuation-compensated Airy light-sheet microscope setup

Our two-photon attenuation-compensated Airy LSFM is based on the OpenSPIM design^17^ in a digitally scanned light-sheet microscope (DSLM) configuration^18^ as shown in Fig. 1(a). The Ti:Sapphire ultrashort pulsed laser (Coherent Chameleon Ultra II, central wavelength 810 nm, 140 fs pulse duration, 80 MHz repetition rate) is spatially filtered and expanded before being directed onto a spatial light modulator (SLM; Hamamatsu, LCOS X10468-04) or cubic phase mask (CPM; PowerPhotonic, custom LightForge mask) to generate the phase profile required to generate a 2+1D Airy profile^12,19^. The SLM (CPM) is then imaged onto a galvo mirror (GM; Thorlabs GVS001) by lenses L1 and L2 and then onto the back aperture of the illumination objective (O1; Nikon, N10XW-PF, 0.30/10x, water immersion) by lenses L3 and L4.

Attenuation-compensation can be implemented on the SLM or by the addition of the compensation module (red dashed box in Fig. 1(a)). The compensation module comprises a linear graded neutral density filter (NDF; Thorlabs, NDL-25C-4, Optical density: 0.04–4.0) and two cylindrical telescopes oriented to elongate the beam along the $z-$axis (CL1, LJ1810L1-B, f = 25 mm, Thorlabs and CL2, LJ1567RM-B, f = 100 mm, Thorlabs) incident onto the NDF and then contract it to the original beam dimensions (CL3, LJ1567RM-B, f = 100 mm, Thorlabs and CL4, LJ1810L1-B, f = 25 mm, Thorlabs). The NDF has a neutral density which varies linearly along its length, therefore the transmission through the NDF varies exponentially along its length. The focal lengths of the cylindrical lenses were chosen to give magnifications of 4x and 0.25x respectively. The compensation module is positioned such that there is an imaging relation between the NDF and the SLM/CPM along the $z-$axis. Different sizes of beam incident on the NDF will yield different intensity (amplitude) gradients across the beam and therefore achieve different strengths of intensity modulation along the beam. For our system parameters (NA_*ill*_ = 0.24, $\alpha =7$) the compensation achieved with the NDF closely matches the SLM-based compensation with $\sigma =0.5$ (Fig. 1(b–e)). Note that for the implementation of SLM-based attenuation-compensation, the phase modulation on SLM was calibrated experimentally, and a linear amplitude modulation was assumed.

The detection arm of the microscope is standard for LSFM. Objective O2 (Olympus, UMPLFLN, 0.50/20x, water immersion) images the light-sheet plane onto an sCMOS camera (CAM; Hamamatsu, C13440-20CU, ORCA-Flash4.0) via a tube lens (TL), excitation filter (FF01-790/SP-25, Semrock), and fluorescence filter (FF01-520/60-25, Semrock). The field-of-view (FOV) of the camera is $300\times 300\,\mu m$.

The single-photon attenuation-compensated Airy LSFM is constructed by substituting the femtosecond laser source for a continuous wave diode laser (Vortran Stradus, $\lambda =488$ nm, 150 mW). We used a 1D CPM to generate a 1+1D Airy beam^12,19^ (either a 1D or 2D CPM could be used) and all lenses were replaced with anti-reflection coated versions optimised for 488 nm. The open-source image acquisition software µManager^20^ was used for acquiring 3D Airy LSFM image stacks of phantoms and biological specimen and the subsequent data processing.

### Fluorescent attenuating phantoms

For testing the performance of two-photon attenuation-compensating Airy LSM, green fluorescent beads (400 nm in diameter, Thermo Fisher Scientific, G400) were added to 4 mM NIR dye (American Dye Source, Inc., ADS795WS, absorption coefficient: $1.6\times {10}^{5}$ L mol^−1^ cm^−1^) and later mixed with an equal volume of 1% low-melting point agarose to make a strongly attenuating (absorbing) phantom. This suspension was injected into an FEP tube (Adtech Polymer Engineering Ltd., FT1.3 × 1.6), which was sealed with putty (Hawksley, Crystaseal) at both ends, and imaged under the microscope. The final concentration of NIR dye in the phantom was 2 mM.

We made strongly attenuating phantoms for single-photon attenuation-compensating Airy LSM by adding fluorescent red beads (2 m in diameter, Thermo Fisher Scientific, R0200) to high concentration fluorescein dye (1.76 mM, Thermo Fisher Scientific, FITC) and mixing this with an equal volume of 1% low-melting point agarose.

### Spheroids

To generate cellular Spheroids, we used HEK-293 T17 and SH-SY5Y cells stably expressing GFP. A variable number of cells (e.g. 500, 1000, 2000) depending on the wanted spheroids size were plated in ultra-low attachment 96- well round bottom cell culture plates (Corning Costar 7007). After a 48 hours period (or when they have reached the desired size) the spheroids were fixed in PFA. The samples were then embedded in 1% low-melting point agarose and mounted in FEP tubes for the imaging procedure.

## Supplementary information

Supplementary Information.

## Data Availability

The research data and materials supporting this publication can be accessed at 10.17630/5d8d3500-cd31-4776-8c01-57ad3507d7ff.
