# Supplementary material for: Multi-photon attenuation-compensated light-sheet fluorescence microscopy
Source: Sci Rep. 2020 May 15;10:8090. doi: 10.1038/s41598-020-64891-8 (PMC7229186; doi:10.1038/s41598-020-64891-8)
Supplement: Supplementary file 1 — Supplementary Information. [file 41598_2020_64891_MOESM1_ESM.pdf]

Multi-photon attenuation-compensated light-sheet fluorescence microscopy: supplementary material

Madhu Veettikazhy[1,*], Jonathan Nylk[2], Federico Gasparoli[2], Adrià Escobet-Montalbán[2], Anders Kragh Hansen[3], Dominik Marti[1], Peter Eskil Andersen[1], and Kishan Dholakia[2,4]

[1]DTU Health Tech, Technical University of Denmark, Frederiksborgvej 399, 4000 Roskilde, Denmark
[2]SUPA, School of Physics and Astronomy, University of St Andrews, North Haugh, St Andrews, KY16 9SS, UK
[3]DTU Fotonik, Technical University of Denmark, Frederiksborgvej 399, 4000 Roskilde, Denmark
[4]Department of Physics, College of Science, Yonsei University, Seoul 03722, South Korea
[*]Corresponding author: madve@dtu.dk

ABSTRACT

This document provides supplementary information to "Multi-photon attenuation-compensated light-sheet fluorescence microscopy." This supplementary document consists of 3 supplementary figures and associated notes.

Supplementary Materials

Supplementary Note 1: Monte Carlo Radiative Transfer Modelling

Absorption and scattering are two major phenomena that impedes deeper penetration of incident optical fields into tissue, but both absorption and single scattering add up to an exponential decay of intensity following the Beer-Lambert law $I/I_0 = \exp(-\mu x)$, where x is the penetration depth and μ is the tissue attenuation coefficient. The ratio of single scattering to multiple scattering reduces with x, and that could have an influence on the attenuation profile of the Airy beam, and subsequently of the light sheet. We employed open-source codebase of MCmatlab [1] for numerically simulating the intensity decay profile of light-field propagating through a standard biological tissue, with varied scattering properties given by the tissue's scattering coefficient μ_s and its Henyey-Greenstein scattering anisotropy factor g. It was observed that the normalized fluence rate exponentially decays against penetration depth over a detection FOV of 300 µm × 300 µm. The MC simulations thus demonstrated that multiple scattering results in negligible deviation from linear attenuation over length scales relevant for microscopy.

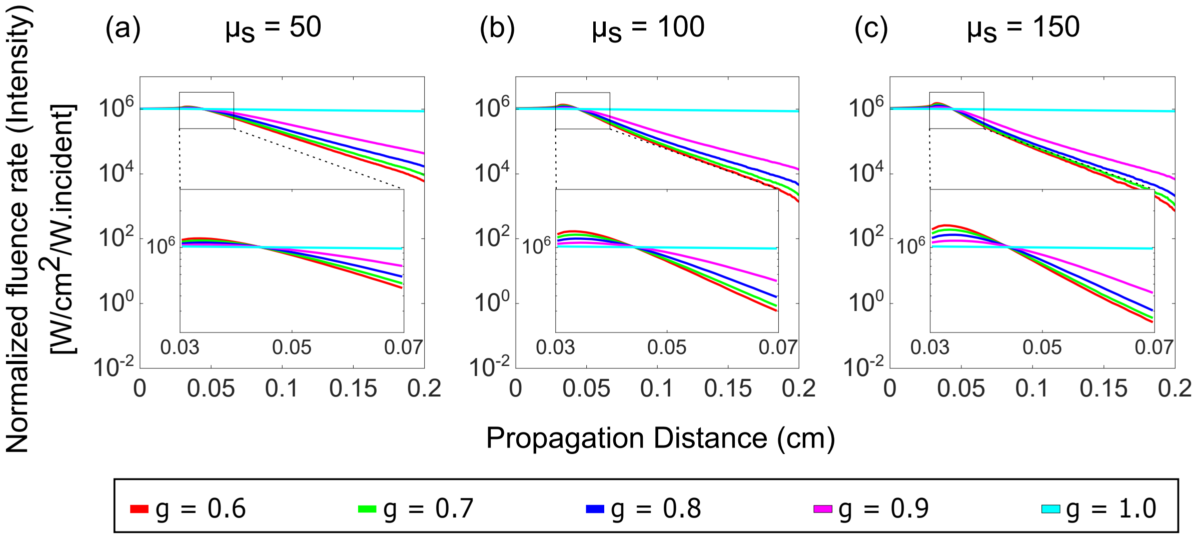

Figure 1. (**a**): Normalized fluence rate per watt of incident light of pencil beam against penetration depth x in tissue with $\mu_s = 50$ and g varying from 0.6 to 1. (**b**): Normalized fluence rate per watt against x in tissue with $\mu_s = 100$ and g varying from 0.6 to 1. (**c**): Normalized fluence rate per watt against x in tissue with $\mu_s = 150$ and g varying from 0.6 to 1. The range of interest 0.03 cm-0.07 cm (400 µm) is highlighted in all the sections.

Figure 1 presents the results of Monte Carlo simulations. Fig. 1 (a) plots normalized fluence rate per watt of incident light of pencil beam against penetration depth x in tissue with $\mu_s = 50$ and g varying from 0.6 to 1. Fig. 1 (b) plots the same for a tissue with $\mu_s = 100$ and g varying from 0.6 to 1, and Fig. 1 (c) corresponds to a tissue with $\mu_s = 150$ and g varying from 0.6 to 1.

Supplementary Note 2: Fourier analysis of Airy LSM images of spheroids

We analysed the 3D image stack of spheroids in the spatial frequency domain and calculated the spectral magnitude within the nth spectral window of the images in y-z plane, $S_n(x)$[2], given by:

$$S_n(x) = \frac{\int_{f_{r,n-1}}^{f_{r,n}} \int_0^{2\pi} |\tilde{I}(f_r, f_\theta; x)| f_r \, df_r \, df_\theta}{\int_{f_{r,n-1}}^{f_{r,n}} \int_0^{2\pi} f_r \, df_r \, df_\theta} \tag{1}$$

where $\tilde{I}(f_r, f_\theta; x)$ is the Fourier transform of the image plane $I(y,z;x)$ in cylindrical coordinates and $f_{r,n}$ is the radial spatial frequency separating spectral windows in 10% increments of the diffraction limit: $f_r, n = (n/10)(2NA/\lambda)$. This analysis segments the Fourier space of images orthogonal to depth (y-z) into rings corresponding to different frequency ranges, and calculates the Fourier content in each ring (see Figs. 2 and 3). We observed that the peaks in Fourier spectra corresponding to signal for rings above 40%-50% are strongly attenuated. Besides, since we are looking for nuclei within spheroids with no high frequency details, we could consider the frequency band of 0%-10% as the local dc component. Thus, we considered signal corresponding to 10%-50% Fourier spectra as "signal" and that corresponding to 80%-100% Fourier spectra as "noise", and calculated signal-to-noise ratio (SNR) for the three attenuation-compensation modalities.

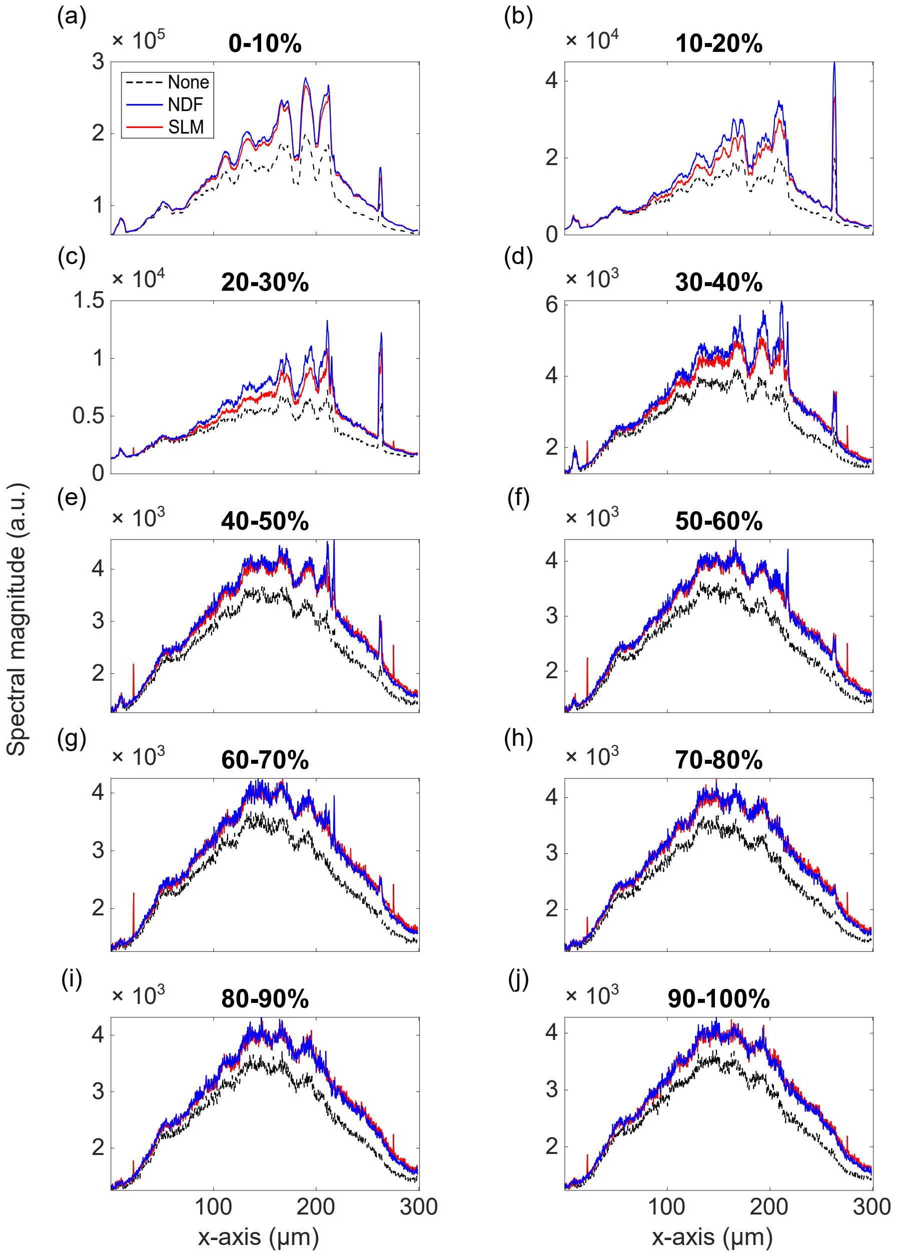

Figure 2. (**a-j**) Plots of spectral magnitude $S_n(x)$ versus tissue depth x for the two-photon Airy LSM recorded image stacks of HEK-293 spheroid stably expressing GFP with no attenuation-compensation, NDF-based, and SLM-based ($\sigma = 0.5$) attenuation compensation.

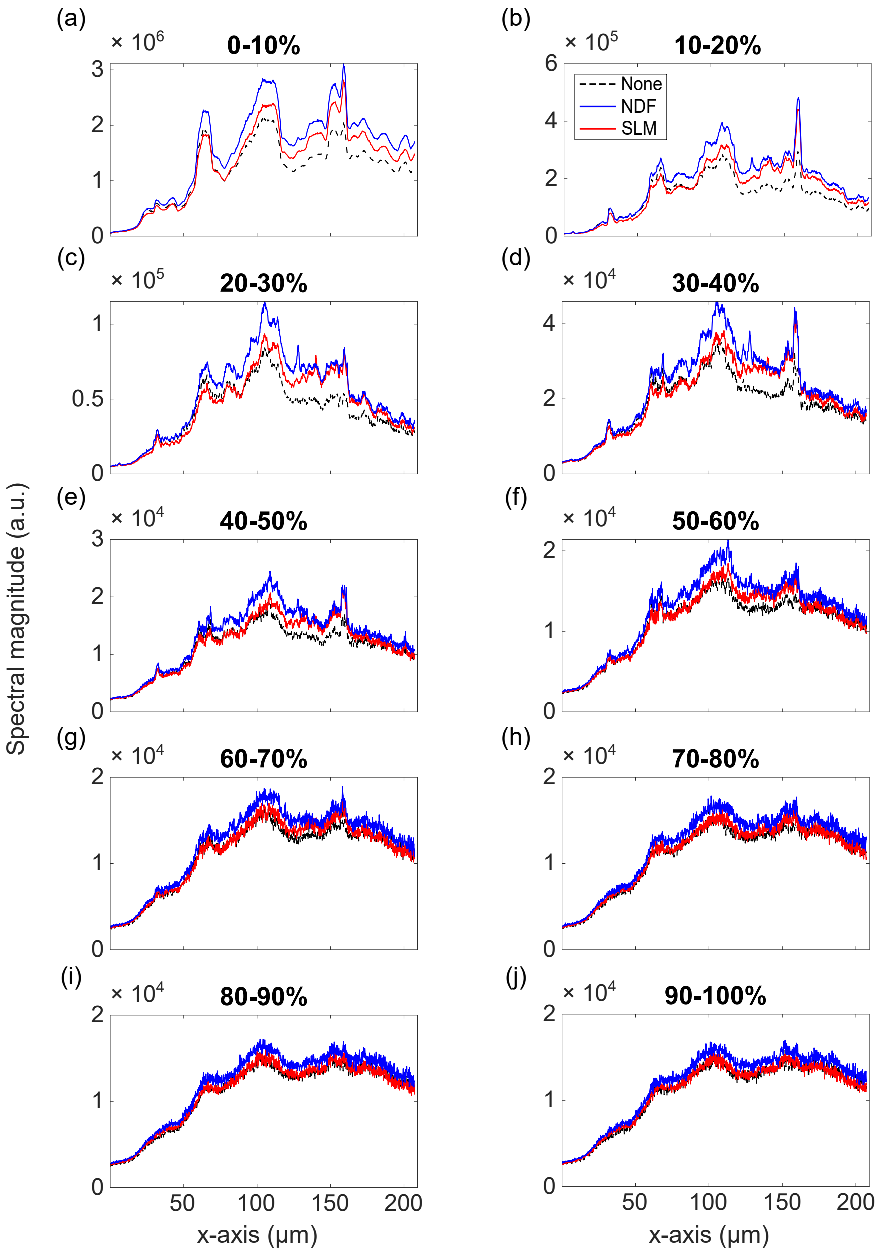

Figure 3. (**a-j**) Plots of spectral magnitude $S_n(x)$ versus tissue depth x for the single-photon Airy LSM deconvolved image stacks of SH-SY5Y spheroid stably expressing GFP with no attenuation-compensation, NDF-based, and SLM-based ($\sigma = 0.8$) attenuation compensation.

References

1. Marti, D., Aasbjerg, R. N., Andersen, P. E. & Hansen, A. K. MCmatlab: an open-source, user-friendly, MATLAB-integrated three-dimensional Monte Carlo light transport solver with heat diffusion and tissue damage. *J. Biomed. Opt.* **23**, 121622, DOI: 10.1117/1.JBO.23.12.121622 (2018).

2. Nylk, J., McCluskey, K., Aggarwal, S., Tello, J. A. & Dholakia, K. Enhancement of image quality and imaging depth with airy light-sheet microscopy in cleared and non-cleared neural tissue. *Biomed. Opt. Express* **7**, 4021–4033, DOI: 10.1364/BOE.7.004021 (2016).
